# Dual-functionalized liposomal delivery system for solid tumors based on RGD and a pH-responsive antimicrobial peptide

**DOI:** 10.1038/srep19800

**Published:** 2016-02-04

**Authors:** Qianyu Zhang, Libao Lu, Li Zhang, Kairong Shi, Xingli Cun, Yuting Yang, Yayuan Liu, Huile Gao, Qin He

**Affiliations:** 1 Key Laboratory of Drug Targeting and Drug Delivery Systems, West China School of Pharmacy, Sichuan University, No. 17, Block 3, Southern RenminRoad, Chengdu 610041, China

## Abstract

[D]-H_6_L_9_, as a pH-responsive anti-microbial peptide (AMP), has been evidenced by us to be an excellent choice in tumor microenvironment-responsive delivery as it could render liposomes responsive to the acidified tumor microenvironment. However, [D]-H_6_L_9_-modified liposomes could not actively target to tumor area. Therefore, integrin α_v_β_3_-targeted peptide RGD was co-modified with [D]-H_6_L_9_ onto liposomes [(R + D)-Lip] for improved tumor delivery efficiency. Under pH 6.3, (R + D)-Lip could be taken up by C26 cells and C26 tumor spheroids (integrin α_v_β_3_-positive) with significantly improved efficiency compared with other groups, which was contributed by both RGD and [D]-H_6_L_9_, while RGD did not increase the cellular uptake performance on MCF-7 cells (integrin α_v_β_3_-negative). Results showed that RGD could decrease cellular uptake of (R + D)-Lip while [D]-H_6_L_9_ could increase it, implying the role of both RGD and [D]-H_6_L_9_ in cellular internalization of (R + D)-Lip. On the other hand, (R + D)-Lip could escape the entrapment of lysosomes. PTX-loaded (R + D)-Lip could further increase the cellular toxicity against C26 cells compared with liposomes modified only with RGD and [D]-H_6_L_9_ respectively, and achieve remarkable tumor inhibition effect on C26 tumor models.

Lowered extracellular pH has been highlighted as one of the major heterogeneity of tumors, which was triggered by the hypoxia and the abnormal tumor metabolic process, leading to the acidification of tumor extracellular environment^1^,^2^,^3^. This unique feature of tumors could be utilized to design pH-responsive platforms. Among them, histidine-based drug delivery systems have received considerable attention^4^,^5^,^6^,^7^. With a pKa around 6.5, the imidazole ring on hisitidine confers the ability to donate or receive protons to histidine^3^, meaning that it could protonate into a positively-charged state under acidic tumor microenvironment yet remain slightly negatively charged in tissues with normal physiological condition. Histidine-rich cell penetrating or membrane-lytic peptides were thus designed^8^,^9^,^10^,^11^,^12^,^13^,^14^, and their indiscriminative and strong penetration in normal tissues could be alleviated; while in acidified environment such as tumors, their membrane-permeation ability and other activity could be restored.

pH-responsive peptides could also be anchored to nano-carriers, bestowing nano-carriers with pH-responsiveness as well^13^,^14^,^15^,^16^,^17^. We have already constructed a pH-responsive anti-microbial peptide (AMP) [D]-H_6_L_9_-mediated PEGylated liposome (D-Lip) in our previous work^16^,^17^ and it showed competence in tumor cellular delivery both *in vitro* and *in vivo*. Although nano-carriers of this kind could be more efficiently internalized by tumor cells under acidified environment, this was realized only by the passive accumulation of surface-PEGylated nano-carriers (liposomes or polymeric micelles) by EPR effect, since these peptides were not tumor-homing peptides with targeting capacity. Therefore, the introduction of proper ligands that could specifically recognize tumors was necessary.

Besides the acidification by aerobic glycolysis in tumors, angiogenesis was another important hallmark of cancers that were ubiquitous in solid tumors, playing important role in tumor growth and metastasis^18^,^19^. Integrin α_v_β_3_ are universally expressed in a myriad of tumors, and could be considered as tumor-specific receptors which could be targeted by circulating ligands^20^, including cyclic RGD^21^. As an excellent tumor-targeting moiety, RGD or RGD-anchored nano-carriers could target tumors with efficiency^22^,^23^,^24^. It could be combined with other ligands or cell penetrating peptides for augmented tumor-targeted delivery in multi-functional nano-carriers^25^,^26^,^27^,^28^,^29^,^30^,^31^. Together with cell penetrating peptides such as TAT, an enhanced photodynamic therapy of Hela cells and Hela tumor-bearing mice could be acquired^27^. When TAT and RGD were co-modified on silica nanoparticles and used for tumor therapy, murine tumor growth could be successfully repressed^28^. However, TAT, as a typical cell penetrating peptide and without shielding from a hydrophilic protection layer such as PEG, could interact directly with blood serums which altered its pharmacokinetic profiles, jeopardizing its *in vivo* application.

In this work, we engineered a dual-functional liposome [donated as (R + D)-Lip], which was co-decorated by a pH-responsive anti-microbial peptide [D]-H_6_L_9_ and a specific ligand RGD for tumor delivery. Before reaching tumors, histidine-rich peptide [D]-H_6_L_9_ was inactivated in blood circulation and normal tissues (pH 7.4). Upon arrival at tumors, RGD as a targeting ligand could identify tumor cells (C26 cells) that expressed integrin α_v_β_3_ receptors in the first place. Then the cell permeation by [D]-H_6_L_9_ could be potentiated in the acidified tumor environment (pH 6.3), boosting tumor-specific delivery of liposome and its cargos. (Fig. 1)

## Results

### Preparation and characterization of liposomes

**Figure 2:**
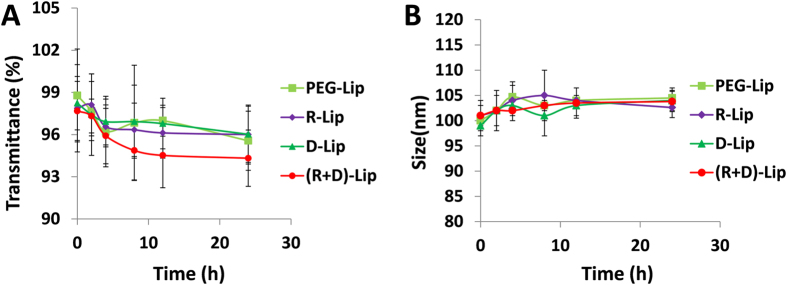
Variations in turbidity (**A**), represented by transmittance) and particle sizes (**B**) of liposomes in 50% FBS in PBS for 24 h under 37 °C. (n = 3, mean ± SD)

Liposomes were prepared with film dispersion method. As could be seen from Tab 1, the sizes of liposomes were all within the range of 115 nm~140 nm, whether under pH 7.4 or pH 6.3, which was suitable for *in vivo* tumor delivery. TEM image and size distribution of PTX-loaded (R + D)-Lip were also displayed in Fig. S1. The entrapment efficiency of PTX by different liposomes all reached beyond 90%, and the loading capacity was 2.75% (w/w) for (R + D)-Lip. The serum stability of liposomes could also be validated (Fig. 2), with transmittance hardly changed over 24 h, indicating that no aggregation was formed in serum which was due to the PEGylation of liposomes. The most intriguing phenomenon should be the conversion of zeta potentials of [D]-H_6_L_9_-anchored liposomes [including D-lip and (R + D)-Lip] from negative to positive when the pH value was lowered from 7.4 (normal tissue environment-mimicking) to 6.3 (tumor microenvironment-mimicking) (Table 1). This was due to the protonation of histidines in peptide [D]-H_6_L_9_, which has already been evidenced by our previous work^16^,^17^. It could be seen that (R + D)-Lip also displayed charge reversal capacity (Table 1), showing that the addition of RGD onto liposome surface did not influence the pH-responsiveness of peptide [D]-H_6_L_9_.

### *In vitro* cellular uptake assay and cell viability assay

**Figure 3:**
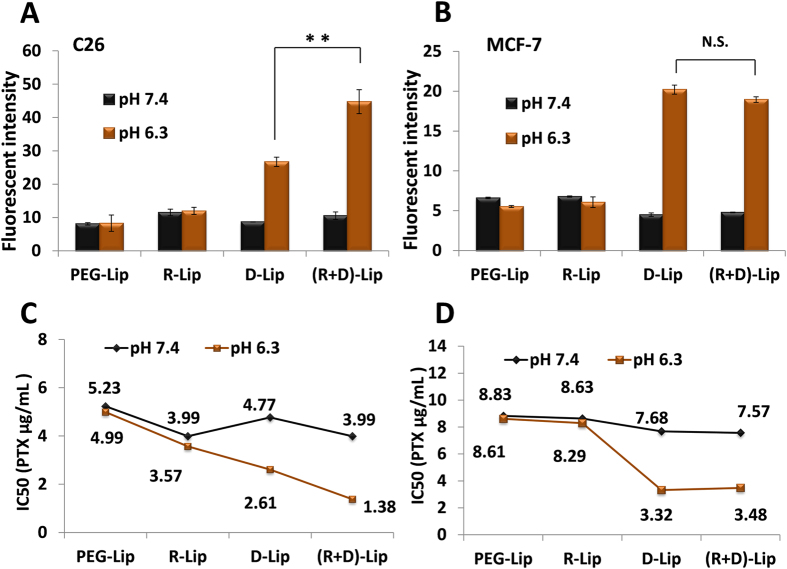
Cellular uptake of liposomes by C26 (**A**) intergin α_V_β_3_-positive cells) and MCF-7 (**B**) intergin α_V_β_3_-negative cells) determined by flow cytometry as well as the IC_50_ values of PTX-loaded liposomes on C26 (**C**) and MCF-7 (**D**) tumor cells determined by MTT assay. ** indicated p < 0.05 and N.S indicated no significant difference. (n = 3, mean ± SD)

Western Blot assay was performed to quantify the expression level of integrin α_v_ and integrin β_3_ on C26 and MCF-7 cells (Fig. S2). According to the results, C26 was selected as the representative α_v_β_3_ integrin receptor-positive tumor cells and MCF-7 was the representative of α_v_β_3_ integrin receptor-negative tumor cells^32^,^33^,^34^. On C26 cells, the cellular uptake of R-Lip was 1.42-fold and 1.45-fold compared to PEG-Lip under pH 7.4 and pH 6.3 respectively, demonstrating the introduction of RGD did facilitate the cellular uptake efficiency of liposomes by α_v_β_3_-positive tumor cells (Fig. 3A). By contrast, this preferential uptake of R-Lip over PEG-Lip did not happen on MCF-7 cells (Fig. 3B). D-Lip showed pH-responsive cellular uptake profile on both C26 cells (3.07-fold increase under pH 6.3 compared to pH 7.4) and MCF-7 cells (4.49-fold increase under pH 6.3 compared to pH 7.4). Similarly, (R + D)-Lip exhibited pH-responsiveness on both cells, although to different degrees: on C26 cells, cellular uptake efficiency increased by 4.25-fold under pH 6.3 compared with pH 7.4, showing that the cellular uptake of (R + D)-Lip by α_v_β_3_-positive tumor cells such C26 was mediated by both was contributed by both RGD and [D]-H_6_L_9_. On the contrary, RGD did not increase the cellular uptake of (R + D)-Lip by MCF-7 cells under pH 6.3 compared with D-Lip. As for the cytotoxicity of PTX-loaded liposomes assessed by MTT assay, it could also be concluded that the cytotoxicity of PTX-loaded (R + D)-Lip on C26 cells under pH 6.3 was due to the combination of RGD and [D]-H_6_L_9_ (Fig. 3C), while on MCF-7 cells, the presence of RGD on the surface of liposomes did not promote cytotoxicity of PTX-loaded liposomes (Fig. 3D). Detailed cellular inhibition rates of different liposomes could be found in Tables S1 and S2.

### Effect of free peptide solution on cellular uptake and subcellular localization

**Figure 4:**
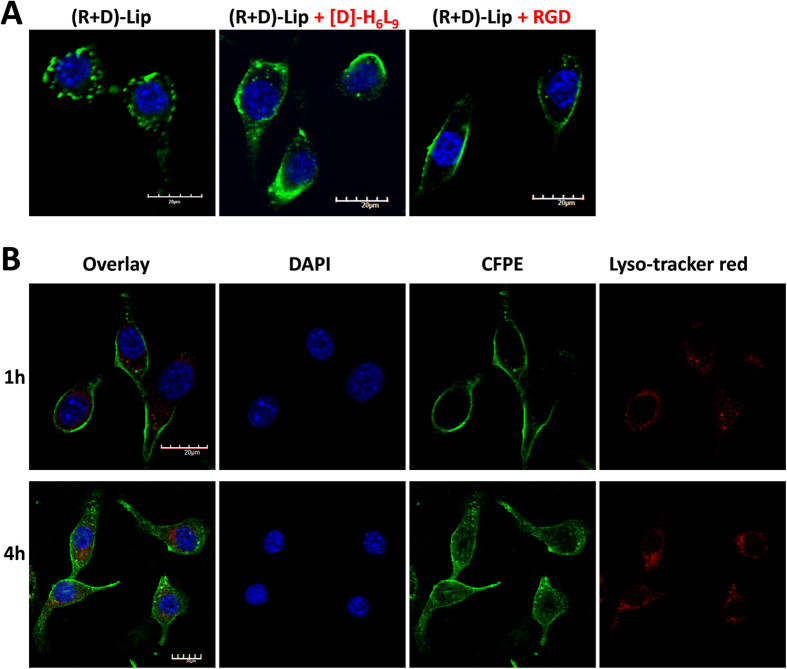
(**A**) CLSM images of C26 cells that have been incubated with CFPE-labeled (R + D)-Lip in the presence of free RGD peptide or [D]-H_6_L_9_. (**B**) Lysosome staining after C26 cells were treated with CFPE-labeled (R + D)-Lip for 1 or 4 h. Scale bar represents 20 μm.

The competitive inhibition assay was to discern the impact of excessive free [D]-H_6_L_9_ or RGD peptide on cellular uptake of (R + D)-Lip. After 2 h, free [D]-H_6_L_9_ turned to somewhat promote the cellular uptake of (R + D)-Lip, while it was notable that free RGD could strongly suppress the cellular endocytosis process (Fig. 4A).

It was shown in Fig. 4B that within 1 h, CFPE-labeled (R + D)-Lip was mostly adjacent to cellular membrane and distributed within cellular periphery. Liposomes with positive charged surface (both cationic liposomes and cationic CPP-mediated liposomes) were often found within the peripheral part of cells after a short period of cellular uptake^15^,^35^,^36^. After 4 h, C26 cells could readily take up (R + D)-Lip. Although there was some overlap of lysosomes and liposomes (shown in color yellow), the fluorescence of most (R + D)-Lip was found to be outside the lysosomes (Fig. 4B). This should be owing to the presence of [D]-H_6_L_9_ on the liposomes as we have evidenced in our previous work^16^,^17^, that [D]-H_6_L_9_ containing liposomes could escape or evade the entrapment of lysosomes.

### Tumor spheroid uptake assay

**Figure 5:**
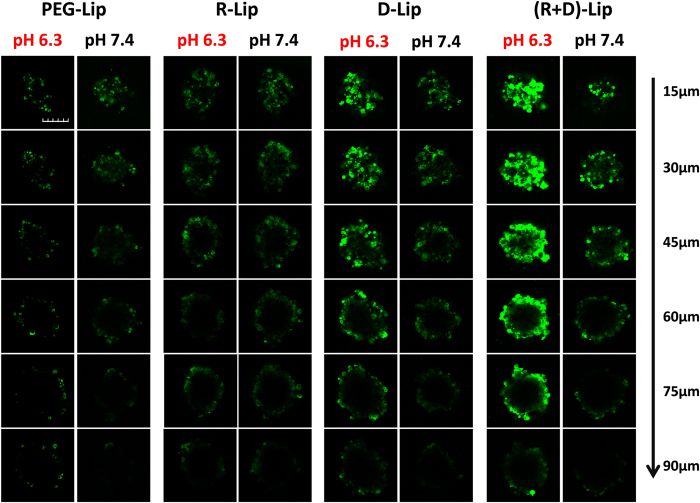
CLSM images showing the uptake of CFPE-labeled liposomes on C26 tumor spheroids after 2 h. Scale bar represented 150 μm.

Similar to the results of cellular uptake in 3.2, D-Lip and (R + D)-Lip exhibited improved tumor spheroid uptake under pH 6.3 compared with 7.4 (Fig. 5). It was noteworthy that under pH 6.3, (R + D)-Lip seemed to be able to accumulate more into the spheroids compared with other groups. This should be attributed to the combined mediation of [D]-H_6_L_9_ and RGD.

### *In vivo* and ex vivo biodistribution

**Figure 6:**
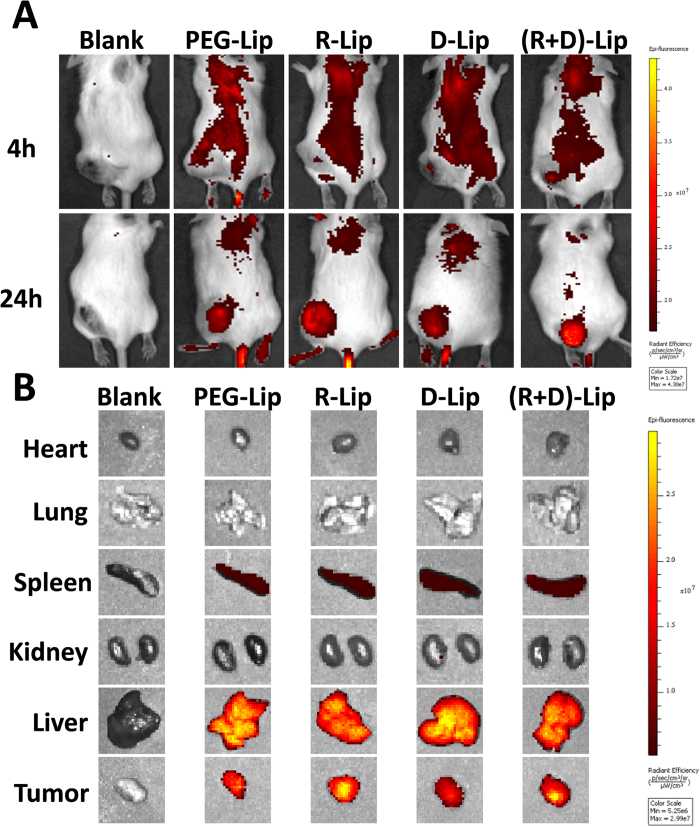
(**A**) *In vivo* images of C26 tumor-bearing Balb/C mice 4 or 24 h after injection of DiR-labeled liposomes. (**B**) Ex vivo images of organs or tumor tissues from C26 tumor-bearing Balb/C mice 24 after injection of DiR-labeled liposomes.

**Figure 7:**
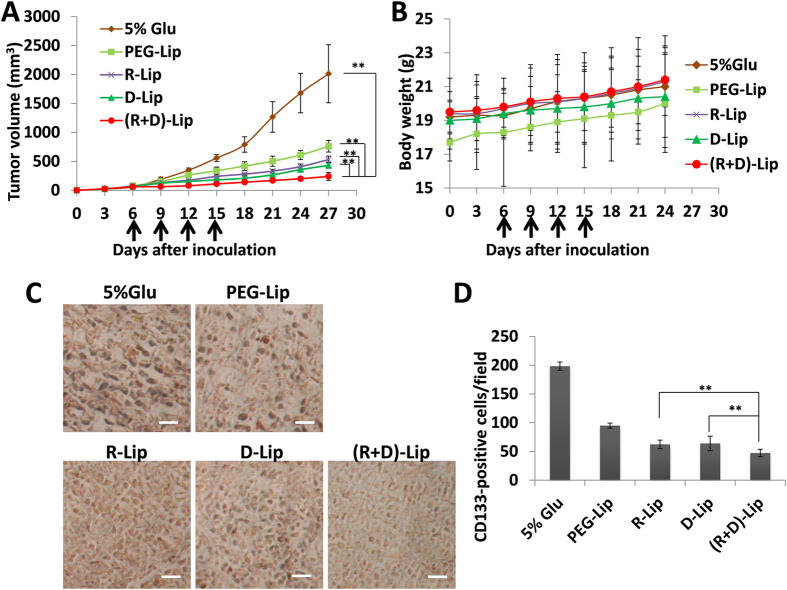
(**A**) Tumor growth curves of mice receiving different PTX-loaded liposomes (n = 6, mean ± SD).Black arrows indicate times of administration. (**B**) Body weight variations of different groups (n = 6, mean ± SD).Black arrows indicate times of administration. (**C**) Representative CD133 immuno-staining (dark brown) of tumor sections. (**D**) Quantification of CD133-positve cells in tumor sections from each group. (n = 6, mean mean ± SD). Images were processed by ImageJ 1.47V. ** indicated p < 0.05.

Due to PEGyalation on liposome surfaces, all four groups of liposomes could reach tumors with efficiency (Fig. 6). As time prolonged from 4 h to 24 h, DiR-labeled liposomes started to accumulate into tumor area (Fig. 6A), and this passive accumulation was mainly due to the EPR effect. Ex vivo fluorescent images of different organs were also captured (Fig. 7B). It showed that the distribution of liposomes in hearts, lungs and kidneys were quite minimal, and their distribution in spleens and livers were quite similar to each other. However, liposomes modified with RGD [including R-Lip and (R + D)-Lip] appeared to be able to accumulate more into tumor area compared with the other two groups (PEG-Lip and D-Lip). The observation on the tumor cyro-sections demonstrated that (R + D)-Lip could be taken up more readily than all the other groups, which might result from the promotion of both [D]-H_6_L_9_ and RGD (Fig. S3).

### Therapeutic evaluation

Compared to all the other groups, the dual-functional liposome (R + D)-Lip could result in more significant tumor growth suppression, and the body weights of all groups hardly dropped during the treatment, implying that all the PTX-loaded liposomes showed little *in vivo* toxicity (Fig. 7A,B). High CD133 expression was considered as one of the typical features of stem cells including some cancer stem cells (CSCs). Therefore, we also assessed the CD133 expression level of different tumor by immunohistochemical staining. It was found out that the CD133-positive cells from the sections of the (R + D)-Lip group appeared to be the least among all the groups (Fig. 7C,D).

## Discussion

Over decades of development, nano-carriers for tumors have evolved from simple and inert drug vehicles to drug delivery platforms which were tumor-targeted or highly tumor microenvironment-responsive. Therefore, a dual-functionalized liposomal delivery system co-modified by RGD and pH-responsive AMP [D]-H_6_L_9_ has been devised by us [(R + D)-Lip]. Due to the presence of [D]-H_6_L_9_, the zeta potential of both D-Lip and (R + D)-Lip could be reversed from negative to positive when pH dropped from 7.4 to 6.3 owing to the protonation of histidine. This in turn led to the significantly improved cellular uptake efficiency on both C26 cells and MCF-7 cells under pH 6.3. However, the introduction of RGD further improved the cellular uptake efficiency of (R + D)-Lip on C26 cells, which was considered as the representative α_v_β_3_ integrin receptor-positive tumor cells. The same phenomenon was not observed on MCF-7 cells, on which the expression of α_v_β_3_ integrin receptor was negative^32^,^33^,^34^.

Meawhile, PTX-loaded (R + D)-Lip could induce the most prominent cellular cytotoxicity under pH 6.3 on C26 cell, implying that the combined effect of both RGD and [D]-H_6_L_9_ could help deliver more liposomes into α_v_β_3_ integrin-positive cells. Multi-functional nano-carriers could draw on the advantages of different targeting ligands, achieving a much more efficient targeted delivery than liposomes with single ligands in synergistic or combined manner^37^,^38^,^39^,^40^. Among them, nano-carriers modified with one specific ligand and one cell penetrating peptides have attracted considerable attention^25^,^39^,^40^. Specific ligand-mediated intracellular delivery was often not efficient enough owing to the fact that receptor-dependent pathway mediated by specific ligand was often saturated^25^, therefore the addition of CPPs could greatly improve cellular delivery. [D]-H_6_L_9_, as a cellular membrane-permeable peptide, acted as the role of a pH-responsive cell penetrating peptide in our work.

It has been reported that α_v_β_3_ integrin receptor-dependent endocytosis was ligand-specific and saturable^25^,^31^, therefore excessive RGD peptide could block the α_v_β_3_ receptor beforehand, resulting in decreased internalization of (R + D)-Lip. As for free [D]-H_6_L_9_, although apparently there was no specific receptor for it on C26 cells, as an AMP which could disrupt membrane integrity in the membrane-depolarizing lytic mechanism, could possibly destabilize and permeate cellular membrane when applied under higher concentration^9^, thus facilitating the intracellular transport of (R + D)-Lip.

Receptor-dependent (in our case, α_v_β_3_ integrin receptor-dependent) endocytosis was often related with endo/lysosomes^41^,^42^, however, our results proved that (R + D)-Lip hold the potential to escape the confinement of lysosomes. This was contributed by peptide [D]-H_6_L_9_, which could cause strong membrane destabilization under endo/lysosome environment, releasing liposomes from endo/lysosomes, which was beneficial for intracellular drug delivery as we have proved before^16^.

Tumor spheroids were favorable and simple models for tumor chemotherapy evaluation^43^. It could in a way predict the diffusion-based transport of nanoparticles in a milieu that better reflects the structural and microenvironmental heterogeneity commonly associated with solid tumors^44^. RGD was known for its solid tumor penetration capacity which was mediated by integrin-dependent transcytosis into deeper tumor issues^45^,^46^. Together with [D]-H_6_L_9_, (R + D)-Lip appeared to be able to permeate into the inner area of tumor spheroids. It has been reported that hypoxia areas and pH gradients exists within tumor spheroids and the core could be further acidified thereafter^47^. Therefore theoretically, it was possible that [D]-H_6_L_9_ could be activated in the core with lowered pH and (R + D)-Lip was able to penetrate into and stay in tumor cells within the deeper area in tumor spheroids compared to other groups. In this work, R-Lip did not seem to exhibit significant penetration on spheroids, probably because the uptake of CFPE-labeled liposomes by spheroids remained on a lower level compared to D-Lip and (R + D)-Lip (under pH 6.3) and the penetration capacity was not as excellent as (R + D)-Lip in an *in vitro* model. The biodistribution of different liposomes and their *in vivo* property should be further investigated.

As indicated by our previous work, [D]-H_6_L_9_ did not possess active tumor-homing capacity^16^,^17^. Meanwhile, it has been confirmed that RGD could be considered as tumor-homing ligand and applied in active targeting of various tumors for altered and enhanced tumor accumulation^48^,^49^,^50^. *In vivo*, RGD could bind with tumor blood vessel or even tumor cells that overexpressed α_v_β_3_ integrin receptor to realize active tumor targeting^25^,^51^. It has been reported that vascular targeting by RGD-functionalized nano-carriers was feasible, which resulted in rapid and efficient early binding to tumor blood vessels. Overtime, passive targeting (EPR effect) stepped in and was more efficient by solely vascular targeting, and in return led to higher overall tumor retention levels^52^. This accounted for the increased accumulation of R-Lip and (R + D)-Lip in tumors. This meant that (R + D)-Lip that we have constructed in this work owned distinct advantages over the D-Lip we have previously constructed.

CD133 expression was a very typical feature of stem cells, and has been identified as a special marker for cancer stem cells (CSCs)^53^. CSCs were considered to be resistant to chemotherapy, and some studies suggested that CSCs were held as the culprit of tumor metastasis and relapse^54^. Therefore, successful reduction and even eradication of cancer tumor cells would be of potential significance to tumor therapy. Results from Fig. 7C showed that the amount of CD133-positive CSCs from group (R + D)-Lip was minimal among all. It could be concluded from Fig. 5 that (R + D)-Lip could penetrate deeper into the tumor spheroids, and more (R + D)-Lip could be taken up and retained by tumor spheroids than other groups. Combing the results together, we might make a bold speculation that due to the fact that more PTX-loaded (R + D)-Lip could be delivered into tumors, the tumor treatment outcome for PTX-loaded (R + D)-Lip was much better than other groups, which indicated certain significance to tumor therapy. Therefore, (R + D)-Lip proved itself as an outstanding dual-functionalized drug delivery platform for tumor delivery, especially for α_v_β_3_ integrin-positive tumor cells with significantly improved tumor therapy effect.

## Methods

### Materials

SPC (soybean lecithin) was purchased from Shanghai Taiwei Chemical Company (Shanghai, China). Cholesterol was purchased from Kelong Chemical Company (Chengdu, China). DSPE-PEG_2000_, and 2-dioleoyl-sn-glycero-3- phosphoethanolamine-N-(carboxyfluorescein) (CFPE) were purchased from Avanti Polar Lipids (Alabaster, AL, USA). DSPE-PEG_2000_-Mal was purchased from Shanghai Advanced Vehicle Technology (AVT) L.T.D. Company (Shanghai, China). cRGDfK-cysteine peptide (cycle RGDfK-Cys) and [D]-H_6_L_9_ peptide with a terminal cysteine (LHLLHHLLHHLLHLL-Cys, the underlined letters were D-amino acids) were synthesized according to the standard solid phase peptide synthesis by ChinaPeptides Co. Ltd. (Shanghai, China). 1, 10- Dioctadecyl- 3, 3, 30, 30 - tetramethylindotricarbocyanine iodide (DiR)was purchased from Biotium. Lysotracker ^TM^ was obtained from Invitrogen (Carlsbad, CA, USA). 6-diamidino-2-pheylindole (DAPI) and was purchased from Beyotime Institute Biotechnology (Haimen, China). Other chemicals and reagents were of analytical grade. DSPE-PEG_2000_-RGD and DSPE-PEG_2000_-[D]-H_6_L_9_ were synthesized and purified according to our previously reported methods^16^,^31^.

C26 cells (murine colon cancer cells) and MCF-7 cells () were cultured in RPMI-1640 medium (GIBCO) supplemented with 10% FBS at 37 °C in a humidified 5% CO_2_ atmosphere. Plastic cell culture dishes and plates were purchased from Wuxi NEST biotechnology Co. (Wuxi, China). BALB/C mice purchased from experiment animal center of Sichuan University (P.R. China). All animal experiments were performed in accordance with the principles of care and use of laboratory animals and were approved by the experiment animal administrative committee of Sichuan University. 6-week to 8-week old Balb/C mice were inoculated with 5 × 10^5^ cells subcutaneously into the left flank. Tumors were allowed to grow to an average volume of 50–100 mm^3^ (tumor volume = length × width^2^ × 0.52) for the biodistrubition study.

### Preparation and characterization of liposomes

For the preparation of [D]-H_6_L_9_-anchored liposomes (D-Lip), SPC, cholesterol, DSPE-PEG_2000_ and DSPE-PEG_2000_-[D]-H_6_L_9_ with a molar ratio of 55: 33: 6: 6 were dissolved in the mixture of chloroform and methanol. RGD-anchored liposomes (R-Lip) were prepared with the above materials with DSPE-PEG_2000_-[D]-H_6_L_9_ replaced with DSPE-PEG_2000_-RGD at a molar ratio of 55: 33: 6: 6. [D]-H_6_L_9_ and RGD co-modified liposomes [denoted as (D + R)-Lip] were prepared with SPC, cholesterol, DSPE-PEG_2000_-[D]-H_6_L_9_ and DSPE-PEG_2000_-RGD with a molar ratio of 55: 33: 6: 6. The PEGylated liposomes (PEG-Lip) were prepared with SPC, cholesterol and DSPE-PEG_2000_ at a molar ratio of 57: 33: 10.The organic solvents were removed by rotary evaporation, and a thin lipid film was formed and further dried under vacuum overnight for the removal of remaining solvents. The lipid film was hydrated with appropriate volume of 5% glucose solution and incubated under 37 °C with shaking for 30 min. The obtained liposomes were further dispersed by being intermittently sonicated by a probe sonicator. CFPE, DiR and paclitaxel (PTX) were added to the lipid solution respectively to form the fluorescently labeled or drug-loaded liposomes. Mean particle sizes and zeta potentials of liposomes were measured by Malvern Zetasizer Nano ZS90 instrument (Malvern Instruments Ltd., U.K.). The entrapment efficiency (EE%) of PTX was determined by HPLC.

In order to characterize the serum stability of liposomes, variations in turbidity of liposomes in serum were monitored (Thermo Scientific Varioskan Flash, USA). 100 μL liposomes were mixed with 100 μL fetal bovine serum (FBS) under 37 °C with mild oscillation, and the turbidity was read at each predetermined time points.

### *In vitro* cellular uptake assay

C26 cells were planted at a density of 1 × 10^5^ cells/well into 6-well plates and allowed to grow for 24 h. CFPE-labeled liposomes were applied in fresh cell culture medium, and liposome-free culture medium was considered as the control. Two hours after incubation under 37 °C, cells were washed with cold PBS for three times and then trypsinized and resuspended in 0.4 mL PBS. The fluorescent intensity of cells was measured by a by a flow cytometer (Cytomics^TM^ FC 500, Beckman Coulter, Miami, FL,USA) with the excitation wavelength at 495 nm and the emission wavelength at 515 nm. Ten thousand events were recorded for each sample.

### Cell viability assay

5 × 10^3^ C26 cells were seeded into each well of a 96-well plate. After attachment, cell culture medium was evacuated and liposome-containing medium was added to each well. Twenty four hours later, cytotoxicity was evaluated with MTT assay, with the absorbance read at 570 nm. Non-treated cells were used as controls. All the measurements were repeated in triplicates.

### Effect of free peptide solutions on cellular uptake

C26 cells were seeded onto gelatin-coated cover slip at a density of 2 × 10^4^ cells/well in a 6-well plate. Twenty-four hours later, cells were treated with free [D]-H_6_L_9_ peptide or RGD peptide-containing cell culture medium for 1 h. Then CFPE-labeled (R + D)-Lip were added to cell culture medium and the incubation continued for another 2 h under 37 °C. The cells were washed and fixed with 4% paraformaldehyde and nuclei were stained with DAPI for 5 min followed by washing with cold PBS for 3 times. Coverslips were mounted cell-side down and viewed by confocal microscopy (FV1000, Olympus, USA).

### Subcellular localization

For subcellular localization study, C26 cells were seeded onto gelatin-coated cover slip at a density of 2 × 10^4^ cells/well. After 24 h, cells were applied with CFPE-labeled (R + D)-Lip in cell culture medium and incubated for 0.5 h or 4 h. By the end of incubation, lysotracker red (50 nM) was added into each well and incubated for 30 min. Cells were then washed rapidly with ice cold PBS and fixed with 4% paraformaldehyde at room temperature for 15 min and the nuclei were stained with DAPI for 5 min, and the cells were for visualization (FV1000, Olympus, USA).

### Tumor spheroid uptake assay

C26 cells were trysinized and resuspended in 1640 cell culture medium at a density of 2 × 10^3^ cells/100 μL, and were added to 2% agarose-coated 96-well plate. The formation of C26 tumor spheroids was monitored by optical microscope. Then the spheroids were incubated with CFPE-labeled liposomes for 2 h, gently washed with cold PBS by pipetting, and were fixed in 4% paraformaldehyde under room temperature. Fluorescent images were taken under confocal microscope (FV1000, Olympus, USA).

### *In vivo* and ex vivo biodistribution

C26 tumor-bearing Balb/C mice were randomly divided into different groups with 3 mice each. For the *in vivo* and ex vivo imaging, DiR-loaded liposomes were intravenously injected at a dose of 200 μg DiR/kg. Twenty four hours after injection, mice were imaged with with IVI^®^ Spectrum system (Caliper, Hopkington, MA, USA). Then the mice the executed with cervical dislocation and vital organs and tissues (including hearts, livers, spleens, lungs, kidneys and tumors) were taken out and also imaged. For the cyro-section observation, mice were injected with DiD-labeled liposomes. Twenty four hours later, mice were sacrificed with heart perfusion of saline. Tumors were harvested and cyro-sectioned at a thickness of 10 μm, and sections were treated with 4% paraformaldehyde and DAPI, and were imaged under confocal microscope.

### Therapeutic evaluation

Treatment started on the 5^th^ day of tumor inoculation, and mice were assigned into 6 groups (n = 6) and administered with the following six preparations respectively: PBS, PTX solution (Taxol), PEG-Lip/PTX, D-Lip/PTX, R-Lip/PTX and (D + R)-Lip/ PTX. All the preparations were injected through tail veins every 3 days for 6 times, and tumor volumes were monitored. PTX was administered at a dose of 2 mg/kg. Tumors were dissected afterwards, and immersed in 4% paraformaldehyde. Then tumors were cut into sections of 5 μm, and treated by CD133 antibody to detect the expression levels of the stem cell marker CD133.

## Additional Information

**How to cite this article**: Zhang, Q. *et al.* Dual-functionalized liposomal delivery system for solid tumors based on RGD and a pH-responsive antimicrobial peptide. *Sci. Rep.* **6**, 19800; doi: 10.1038/srep19800 (2016).

## Supplementary Material

Supplementary Information

## Figures and Tables

**Figure 1:**
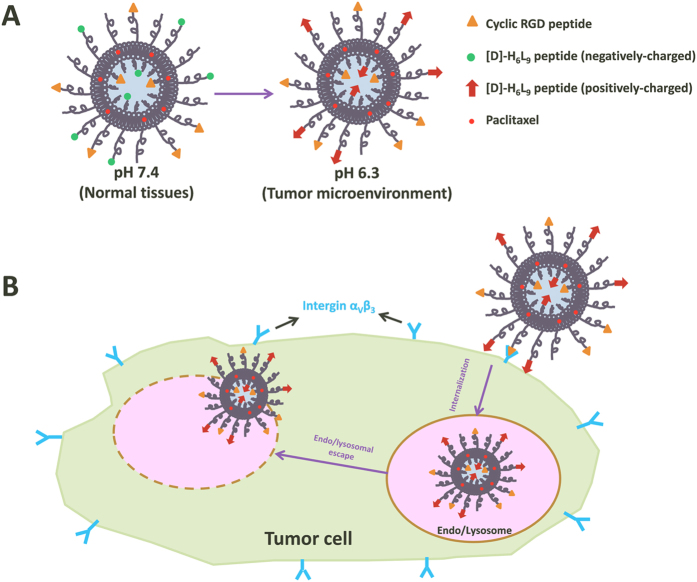
Schematic illustration of (R + D)-Lip. (**A**) The structure of (R + D)-Lip and its surface charge variation under different pH. (**B**) Within tumors, the RGD peptide could recognize intergin α_V_β_3_ that was highly expressed on tumor cells, and [D]-H_6_L_9_ with activated cell penetrating capacity in tumor microenvironment could help deliver (R + D)-Lip intracellularly. This process was contributed by both RGD and [D]-H_6_L_9_, and (R + D)-Lip could escape the entrapment of endo/lysosomes with the aid of the pH-responsive peptide [D]-H_6_L_9_.

**Table 1:** The size and zeta potential measurements of liposomes (n ± 3).

	Size(nm)	PDI	Zeta potential(mV)
*PEG-Lip (pH 7.4)*	120.8 ± 5.6	0.198 ± 0.021	−7.8 ± 1.2
*R-Lip (pH 7.4)*	130.7 ± 6.3	0.199 ± 0.034	−2.5 ± 1.1
*D-Lip (pH 7.4)*	134.1 ± 7.2	0.221 ± 0.023	−8.7 ± 2.2
*(R + D)-Lip (pH 7.4)*	133.3 ± 8.8	0.232 ± 0.025	−6.2 ± 2.0
*PEG-Lip (pH 6.3)*	117.6 ± 7.1	0.188 ± 0.032	−6.3 ± 1.3
*R-Lip (pH 6.3)*	129.3 ± 8.2	0.231 ± 0.022	−4.2 ± 2.1
*D-Lip (pH 6.3)*	135.1 ± 10.2	0.219 ± 0.045	9.9 ± 1.3
*(R + D)-Lip (pH 6.3)*	135.3 ± 9.9	0.235 ± 0.033	8.8 ± 2.1
