# Supplementary material for: Dual-functionalized liposomal delivery system for solid tumors based on RGD and a pH-responsive antimicrobial peptide
Source: Sci Rep. 2016 Feb 4;6:19800. doi: 10.1038/srep19800 (PMC4740748; doi:10.1038/srep19800)
Supplement: Supplementary Information [file srep19800-s1.doc]

**Dual-functionalized liposomal delivery system for solid tumors**

**based on RGD and a pH-responsive antimicrobial peptide**

Qianyu Zhang^*^, Libao Lu^*^, Li Zhang, Kairong Shi, Xingli Cun, Yuting Yang, Yayuan Liu, Huile Gao, Qin He

*Key Laboratory of Drug Targeting and Drug Delivery Systems, West China School of Pharmacy,*

*Sichuan University, No. 17, Block 3, Southern RenminRoad, Chengdu 610041, China.*

*Correspondence and requests for materials should be addressed to Qin He (qinhe@scu.edu.cn).*

**These authors contributed equally to this work.*


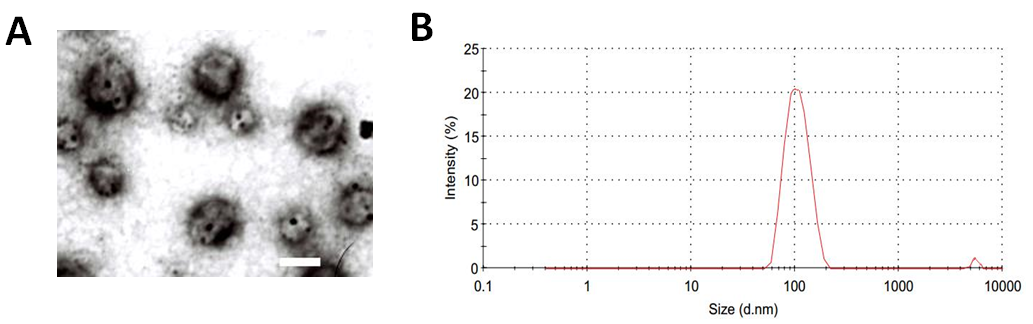


Fig.S1: (A) TEM image (Scale bar represents 100nm) and

(B) size distribution of PTX-loaded (R+D)-Lip


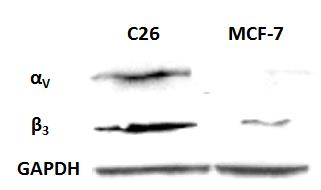


Fig.S2: α_v_ and β_3_ expression levels on C26 cells and MCF-7 cells.


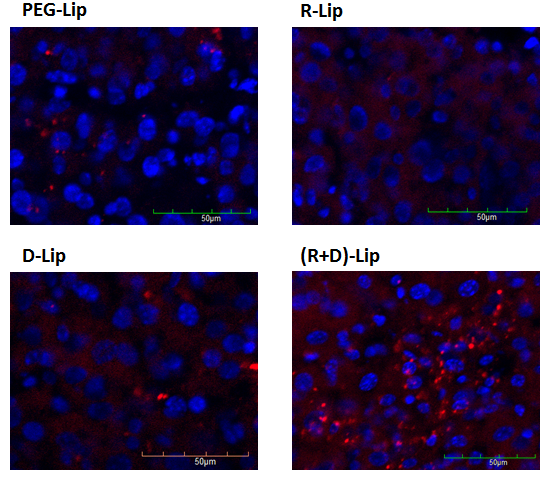


Fig.S3: Representative CLSM images of tumor cyro-sections from C26 tumor-bearing mice receiving DiD-labeled liposomes. Scale bars represent 50μm.

| **PTX concentration (μg/mL)** | **20** | **10** | **5** | **2** | **1** | **0.5** | **0.2** |
| --- | --- | --- | --- | --- | --- | --- | --- |
| PEG-Lip/(pH 7.4) | 29.31±2.86 | 40.22±2.66 | 48.29±3.24 | 63.31±5.23 | 77.18±3.55 | 85.17±6.58 | 92.01±2.32 |
| R-Lip/(pH 7.4) | 22.02±0.32 | 33.33±3.24 | 49.22±5.24 | 61.49±5.71 | 73.65±9.35 | 81.61±6.54 | 90.06±4.34 |
| D-Lip/(pH 7.4) | 26.36±2.44 | 38.63±5.34 | 46.83±2.43 | 63.91±1.54 | 76.53±5.35 | 83.24±9.24 | 93.10±5.75 |
| (R+D)-Lip/(pH 7.4) | 24.13±1.34 | 35.21±0.98 | 47.43±3.55 | 58.49±5.35 | 74.11±6.45 | 79.84±7.01 | 90.39±5.35 |
| PEG-Lip/(pH 6.3) | 27.08±2.34 | 39.91±4.25 | 55.33±5.57 | 63.01±6.28 | 69.84±7.82 | 79.67±6.87 | 90.22±6.66 |
| R-Lip/(pH 6.3) | 24.07±3.44 | 35.13±4.25 | 48.13±3.26 | 58.71±5.25 | 63.24±6.83 | 75.23±8.55 | 92.47±6.46 |
| D-Lip/(pH 6.3) | 20.12±0.55 | 28.04±2.45 | 45.05±5.43 | 53.41±.4.99 | 63.32±7.56 | 71.37±7.66 | 86.75±5.75 |
| (R+D)-Lip/(pH 6.3) | 16.42±2.44 | 25.93±4.44 | 35.92±5.34 | 43.29±6.48 | 50.11±5.77 | 66.31±6.37 | 75.28±6.55 |

Table S1: Inhibition rates of different PTX-loaded liposomes on C26 cells determined by MTT assays. (n=3, mean±SD).

| **PTX concentration (μg/mL)** | **20** | **10** | **5** | **2** | **1** | **0.5** | **0.2** |
| --- | --- | --- | --- | --- | --- | --- | --- |
| PEG-Lip/(pH 7.4) | 37.42±3.22 | 45.82±5.43 | 61.22±6.76 | 73.51±9.35 | 80.86±6.47 | 88.44±9.68 | 94.34±9.23 |
| R-Lip/(pH 7.4) | 36.41±4.43 | 45.01±4.67 | 62.22±9.45 | 72.24±9.57 | 79.32±6.98 | 85.35±7.77 | 90.39±6.35 |
| D-Lip/(pH 7.4) | 35.69±5.54 | 45.01±4.64 | 59.06±6.36 | 67.01±7.98 | 80.32±9.56 | 88.32±9.11 | 96.32±6.09 |
| (R+D)-Lip/(pH 7.4) | 36.33±3.45 | 44.97±4.67 | 57.63±3.57 | 64.33±8.36 | 81.22±5.68 | 89.82±5.36 | 93.32±4.34 |
| PEG-Lip/(pH 6.3) | 36.33±4.52 | 47.42±4.78 | 60.22±4.79 | 71.31±4.22 | 82.32±7.49 | 87.96±9.87 | 95.31±9.56 |
| R-Lip/(pH 6.3) | 39.23±6.36 | 46.27±4.68 | 57.66±8.28 | 67.26±9.45 | 78.63±6.27 | 86.59±7.66 | 93.45±6.94 |
| D-Lip/(pH 6.3) | 25.43±4.35 | 35.22±4.72 | 40.22±5.36 | 52.13±5.73 | 70.75±6.47 | 79.41±6.66 | 87.50±7.53 |
| (R+D)-Lip/(pH 6.3) | 24.76±6.36 | 34.24±3.35 | 43.71±6.37 | 54.73±7.46 | 71.34±5.36 | 80.21±9.71 | 87.22±6.78 |

Table S2: Inhibition rates of different PTX-loaded liposomes on MCF-7 cells determined by MTT assay. (n=3, mean±SD)
